# Stroke Rehabilitation, Novel Technology and the Internet of Medical Things

**DOI:** 10.3390/brainsci16020124

**Published:** 2026-01-24

**Authors:** Ana Costa, Eric Schmalzried, Jing Tong, Brandon Khanyan, Weidong Wang, Zhaosheng Jin, Sergio D. Bergese

**Affiliations:** 1 Department of Anesthesiology, Renaissance School of Medicine, Stony Brook University, Stony Brook, NY 11794, USA; ana.costa@stonybrookmedicine.edu (A.C.); eric.schmalzried@stonybrookmedicine.edu (E.S.); weidong.wang@stonybrookmedicine.edu (W.W.); zhaosheng.jin@stonybrookmedicine.edu (Z.J.); 2 Renaissance School of Medicine, Stony Brook University, Stony Brook, NY 11794, USA; jing.tong@stonybrookmedicine.edu (J.T.); brandon.khanyan@stonybrookmedicine.edu (B.K.)

**Keywords:** neurorehabilitation, stroke rehabilitation, internet of medical things, machine learning, artificial intelligence, telemedicine, wearables, brain–computer interface

## Abstract

Stroke continues to impose an enormous morbidity and mortality burden worldwide. Stroke survivors often incur debilitating consequences that impair motor function, independence in activities of daily living and quality of life. Rehabilitation is a pivotal intervention to minimize disability and promote functional recovery following a stroke. The Internet of Medical Things, a network of connected medical devices, software and health systems that collect, store and analyze health data over the internet, is an emerging resource in neurorehabilitation for stroke survivors. Technologies such as asynchronous transmission to handle intermittent connectivity, edge computing to conserve bandwidth and lengthen device life, functional interoperability across platforms, security mechanisms scalable to resource constraints, and hybrid architectures that combine local processing with cloud synchronization help bridge the digital divide and infrastructure limitations in low-resource environments. This manuscript reviews emerging rehabilitation technologies such as robotic devices, virtual reality, brain–computer interfaces and telerehabilitation in the setting of neurorehabilitation for stroke patients.

## 1. Introduction

Stroke was the third leading cause of disability and death worldwide according to the World Health Organization, with an estimated 93.8 million cases and a global burden of disease of 160 million Disability-Adjusted Life Years, in 2021 [1]. Survivors often experience persistent disability associated with neurological deficits that significantly impair motor abilities, independence in activities of daily living, and overall quality of life. The estimated cost of stroke globally is greater than US$890 billion or 0.66% of the global gross domestic product, with a majority of the global stroke burden (87% of deaths and 89% of disability-adjusted life-years lost) localized to lower-income and lower-middle-income countries [2]. As the population ages, the absolute number of stroke survivors and stroke-related disability continues to increase in the setting of stroke risk factor burden such as metabolic, environmental and behavioral risks.

Rehabilitation plays a central role in mitigating disability and promoting functional recovery across phases of care following a stroke [3]. Stroke rehabilitation focuses on minimizing disability, increasing functional independence and maximizing both physical and mental recovery. A patient’s capacity and stage of stroke recovery (acute, subacute or chronic) influence the intensity and modality of rehabilitation technique [3]. Traditional interventions in stroke rehabilitation include physical, speech and occupational therapies. Physical therapy is traditionally focused on improving physical strength, balance, mobility and coordination through task-oriented exercises, while occupational therapy aims to improve fine motor skills, performance of activities of daily living and adaptive strategies. Traditional rehabilitation strategies require repeated practice and a relatively high intensity compared to the patient’s functional ability. Traditional neurorehabilitation is a physically demanding and repetitive process for the patient, while also being limited by the availability of skilled providers and specialized facilities. Adjunctive and emerging rehabilitation technologies such as telerehabilitation virtual reality (VR), brain–computer interfaces (BCIs) and robotic devices have the potential to improve accessibility, promote patient engagement and refine intervention delivery. This review focuses on the role of novel technology in stroke rehabilitation, and the interconnectivity of devices and interfaces in the Internet of Medical Things (IoMT).

### Methods

We conducted a systematic literature search on PubMed and Medline, for relevant papers published in English. The search terms included “neurorehabilitation”, “telemedicine”, “gamification”, “machine learning”, “robotics”, and “closed-loop”. Article types considered for screening included basic science reports, clinical trials, observational studies, reports/series, systematic reviews, meta-analyses, and review articles. The references were selected for inclusion based on relevance to the topic and quality of evidence. The resulting body of evidence was synthesized into this narrative review article.

## 2. The Internet of Medical Things

The IoMT refers to a network of medical devices, software applications and health systems that are connected through the internet to collect, share, and analyze health data. It enables a health system to connect multiple devices, such as wearable sensors, medical examination monitors, and hospital equipment and infrastructure for creating a digital network. Fast Healthcare Interoperability Resources (FHIR) is an international standard developed by Health Level Seven International (HL7), a not-for-profit international organization composed of health information experts, to enable the secure exchange of electronic health data [4,5]. FHIR defines a modular set of standardized data structures, known as resources, which represent core clinical and administrative concepts such as patients, encounters, diagnostic reports, and medications. These resources are accessed and manipulated through a well-defined application programming interface (API), supporting consistent data exchange across health information systems. Unlike traditional document-centric interoperability models, FHIR emphasizes granular, service-oriented access to discrete data elements, an approach that supports real-time data exchange and promotes reuse of clinical data. Building on earlier HL7 standards, FHIR adopts modern web technologies to reduce implementation complexity and improve scalability, allowing deployment across diverse platforms ranging from enterprise servers to mobile devices. Consequently, FHIR has become a foundational standard for advancing interoperability, patient-centered data access, and innovation within digital health ecosystems.

**Figure 1:**
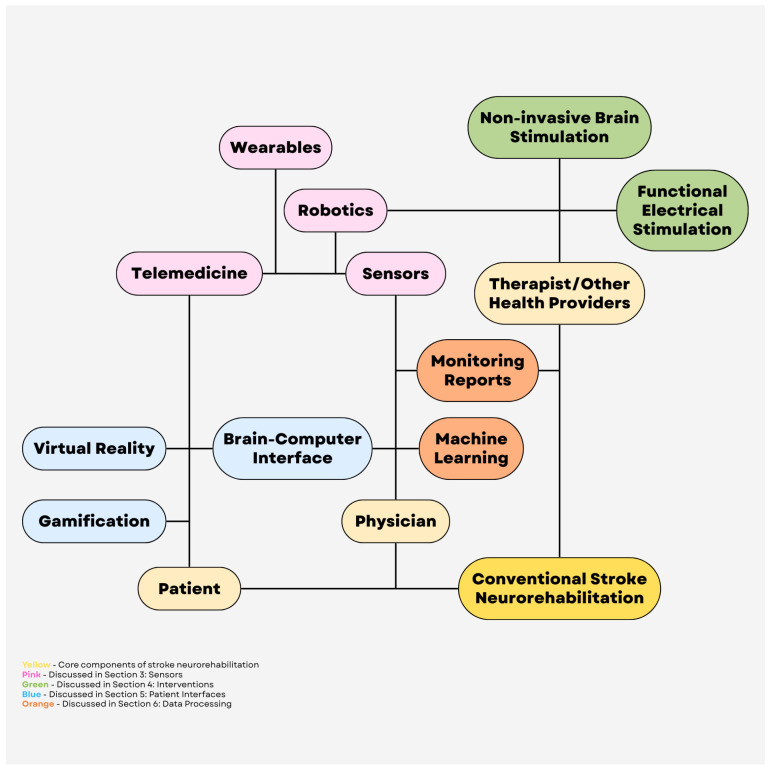
Internet of Medical Things schematic. © Jing Tong via Canva.com.

The IoMT has been utilized in emerging areas of healthcare such as smart hospital systems, remote health monitoring, infectious disease tracking, etc. [6]. The IoMT has a layered architecture, with a perception layer composed of sensors and physical devices, a network layer that provides connectivity, a processing layer that analyzes and stores data, and an application layer that delivers information to end-users such as doctors and patients [7]. Medical devices include wearables, such as fitness trackers, smartwatches, electrocardiograms (ECGs), etc., and implantable devices, such as pacemakers or insulin pumps. Home monitoring systems often utilize glucose monitors, oxygen sensors, ECGs, blood pressure cuffs, etc. Hospital equipment includes infusion pumps, ventilators, and imaging systems that communicate with hospital databases and patients’ electronic health records (EHRs). Medical devices collect patient data, such as heart rate or blood pressure, that is sent to healthcare systems or servers via the internet; artificial intelligence (AI) or algorithms detect problems or trends and then alert patients or healthcare providers if the patient may need further assessment. Figure 1 shows a schematic of the IoMT.

## 3. Sensors

Within the IoMT, sensors constitute the foundational “perception layer” that enables continuous, multi-scale characterization of stroke survivors’ impairments, activity patterns, and physiologic status across inpatient, outpatient, and home environments [8,9,10]. Cameras, wearable devices, and robot-embedded sensors connect to network and application layers, generating high-frequency, multimodal data streams (kinematics, kinetics, physiologic signals, and user interaction metrics) that can be encrypted, aggregated in the cloud, analyzed using advanced analytics or machine learning, and used to inform individualized rehabilitation plans and adaptive interventions [8].

### 3.1. Telemedicine and Diagnostic Use of Videography

High-resolution video integrated into telemedicine platforms has progressed beyond simple videoconferencing to enable structured, quantifiable assessment and therapy delivery. Contemporary telerehabilitation systems can combine synchronous audio–video communication with structured motor or cognitive tasks and cloud-based analytics to approximate or augment in-person examinations [11,12]. In subacute stroke, a post-discharge telerehabilitation program incorporating a personalized rehabilitation plan, regular video consultations, and health education demonstrated superior functional recovery, such as improved activities of daily living, compared with usual care in a randomized trial [13]. Additionally, a multicenter randomized controlled non-inferiority trial found that a self-guided, AI-driven cognitive telerehabilitation program delivered via a mobile platform produced improvements across multiple cognitive measures and was not inferior to therapist-supervised cognitive rehabilitation [12]. In longer-term post-stroke populations, home-based multidomain cognitive training delivered through a VR rehabilitation system has been associated with improvements in cognitive and mood-related outcomes and reduced caregiver burden [14]. For motor rehabilitation, standard video can be paired with markerless computer-vision (pose-estimation) algorithms that track body landmarks to estimate joint angles and movement patterns, enabling remote scoring of gait and upper-limb task performance without dedicated motion-capture hardware [15,16]. When integrated with IoMT platforms that collect and manage the data, these systems support automated data upload, longitudinal tracking, and integration with the EHR [8].

### 3.2. Wearable Sensors

Wearables, such as inertial measurement units (IMUs), pressure sensors, and surface electromyography (EMG) patches, extend measurement beyond the clinic to capture real-world use of the paretic limb, gait quality, cardiovascular load, and engagement with daily activities [9,17,18,19]. Systematic reviews have shown that IMU-based systems can reliably quantify spatiotemporal gait parameters, upper-limb kinematics, and compensatory strategies in stroke survivors, with good agreement with laboratory motion-capture systems and clinical scales [17,19]. Recent studies integrate wearable sensors with machine-learning models to classify task performance, detect compensatory movement patterns, and estimate standardized clinical scores from free-living data, thereby enabling precision rehabilitation dosing and early identification of non-responders [9,18].

Within an IoMT architecture, wearable devices typically communicate with a smartphone or home gateway, which encrypts and transmits data to cloud services for storage and analysis [8,18]. This infrastructure enables near real-time monitoring and automated alerts, such as fall detection, abnormal heart-rate responses to exertion, or sudden reductions in limb use, which can prompt timely outreach or earlier follow-up [19,20]. Wearables can also support stroke rehabilitation by quantifying therapy volume and detecting avoidance of the affected limb even when clinic performance appears adequate [18,20,21]. However, many systems continue to face challenges, including limited battery life and comfort, inconsistent user adherence, lack of validated cut-offs linked to functional outcomes, and fragmented data standards that complicate integration with hospital information systems [8,9,20,21]. Apart from the technical limitations, practical constraints are often the key determinants in adopting wearables. Post-stroke cognitive impairment is common and can undermine adherence to daily wear, device maintenance and charging, and smartphone-guided routines; hemiparesis further increases donning/doffing difficulty and caregiver burden, particularly when multiple sensors are required [22,23,24]. Physical tolerability is also a limiting factor; adhesive electrodes and prolonged skin contact can cause irritation, discomfort, and early discontinuation—issues that may be amplified in older adults and in patients with sensory changes [25].

Finally, greater measurement precision is only clinically actionable when sensor-derived changes are anchored to patient-facing goals. Validation should map wearable metrics (or wearable-predicted clinical scores) to Minimal Clinically Important Differences (MCIDs) and to meaningful real-world functional outcomes (such as activities of daily living), rather than reporting “improvement” that may be statistically significant but clinically trivial [26]. For example, in a randomized feasibility study of a sensor-equipped rehabilitation glove paired with a tablet application, improvement after 14 days of exercises reached or nearly reached MCID estimates, whereas contemporaneous changes in the functional independence measure (FIM), a clinician-validated proxy for day-to-day functional independence, remained well below published MCID thresholds. This finding underscores that measurable sensor-linked improvement does not automatically translate into meaningful change in daily life over short intervals [27]. Addressing these barriers is essential for transitioning wearable technology from research prototypes into routine stroke rehabilitation.

### 3.3. Sensors in Robotic Systems

Rehabilitation robots are equipped with a range of sensors, incorporating joint encoders, force and load sensors, inertial sensors, and, in some cases, EMG or neural interfaces [28,29,30]. These sensors enable precise measurement of interaction forces, movement trajectories, and patient participation, supporting both real-time adaptive assistance and objective outcome tracking [28,29]. IoMT-enabled robots can transmit high-resolution movement and force data to secure cloud platforms, providing remote access to session frequency, intensity, and progression, and enabling therapists or engineers to adjust parameters without onsite presence [30,31]. Recent studies support robot-assisted approaches that incorporate enhanced feedback. For example, a prospective cohort study of a mirror-therapy rehabilitation robot for both upper and lower limbs after stroke found that robot-assisted training was associated with improvements in motor and functional outcomes compared with conventional rehabilitation [32]. Hybrid approaches that combine robot-assisted practice with brain–computer interfaces (BCIs), noninvasive neuromodulation, or functional electrical stimulation (FES) may be particularly effective, as they can more directly link a patient’s movement intent to timed stimulation and the resulting movement, which is thought to better promote use-dependent neuroplasticity [10,28,31,33,34]. As with all measurement instruments, sensor data quality may be affected by improper use, interference and artifacts [35,36]. Prior to integration into clinical decision making and treatment delivery, it is vital to ensure robust product testing, as well as proper training.

## 4. Interventions

In addition to passive monitoring, the IoMT offers an infrastructure for delivering and coordinating technology-enabled neuromodulatory and motor interventions, including non-invasive brain stimulation (NIBS), functional electrical stimulation (FES), and robot-assisted therapy. A common trend across these modalities is the shift toward sensor-driven, performance-adaptive closed-loop systems in which treatment parameters are individualized using patient-specific kinematics, physiologic signals, and adherence data collected in real time and analyzed within cloud-based platforms [8,10,18]. The IoMT can also help address heterogeneity and reproducibility problems in rehabilitation research by capturing objective, time-stamped measures of intervention dose (for example, stimulation parameters, repetitions, assistance levels) and generating longitudinal, sensor-derived profiles (“digital phenotypes”) of recovery over time that add detail alongside standard clinical scales [8,18,37,38].

Closed-loop neurorehabilitation systems use real-time sensor feedback (e.g., EEG/EMG/robot kinematics) to adjust assistance or stimulation parameters, whereas open-loop systems deliver a pre-set assistance/stimulation pattern without adapting to measured response [39,40]. Closed-loop implementations are therefore latency-sensitive; the delay from detected intent to delivered robotic or electrical assistance should be low (nearly instantaneous) and consistent, because tight temporal pairing is critical for activity-dependent learning [41,42]. If intent decoding or control is handled in the cloud, variable network delays can disrupt the intended timing. Studies of teleoperation show that higher latency worsens operator control and that delay/jitter are major bottlenecks in networked robotics [34,39,43]. In practice, the real-time loop (intent detection, safety checks, assistance timing) should run locally (such as on the device itself), while the cloud is used for asynchronous analytics, model updates, and clinician dashboards.

Data hygiene and the black box problem are significant barriers. Even when devices upload session logs, differences in sampling rate, time stamps, calibration, sensor placement, and artifact handling can make the data look precise while being unreliable [37,44]. Proprietary preprocessing and models can further obscure whether a change reflects true recovery versus mis-registration or algorithm drift. IoMT workflows should therefore standardize setup, run basic signal-quality checks, flag missing or low-quality segments, and record which algorithm and software version generated each metric [37,44]. Interoperability is another constraint, as shared data models are essential for clinicians to provide and receive clinical information across platforms and vendors. HL7 FHIR provides interoperability when combined with the IoMT, but adoption is inconsistent [45,46].

### 4.1. Non-Invasive Brain Stimulation

NIBS, primarily repetitive transcranial magnetic stimulation (rTMS) and transcranial direct current stimulation (tDCS), aims to modulate maladaptive network dynamics after stroke, such as interhemispheric imbalance, to enhance training-induced plasticity when paired with task-specific practice [33,47,48,49,50]. Both modalities require specialized knowledge and carry avoidable risks if used incorrectly. rTMS has rare but serious adverse events including seizure risk and requires rigorous screening, trained operators, and protocol adherence. tDCS has a more favorable safety profile but can still cause skin irritation or burns if electrodes are misplaced, contact quality is poor, or safeguards are bypassed [51,52].

Recent research demonstrates modest but clinically relevant effects of rTMS on motor and selected cognitive outcomes, although substantial heterogeneity in effect sizes is observed due to driven by patient factors (stroke phase, baseline severity, lesion, or network integrity) and protocol parameters (site, frequency, intensity, timing relative to training) [33,47,48,49,50]. Evidence from meta-analyses suggests that rTMS benefit is detectable across multiple protocols but remains sensitive to trial design and dosing, highlighting the need for adequately powered, mechanism-informed studies with standardized outcomes and synchronized behavioral training [47,48].

IoMT integration for NIBS is currently most advanced for home-based, remotely supervised-tDCS (RS-tDCS), where compact stimulators can upload session logs (e.g., delivered dose, impedance, and basic safety/tolerability data) and support adherence through tele-supervision and protocol safeguards [53,54,55]. In post-stroke cognitive dysfunction, a randomized trial pairing home-based RS-tDCS with cognitive training demonstrated feasibility and safety, supporting the practicality of moving selected NIBS workflows outside the clinic [53]. Because remote delivery shifts more responsibility to the patient/caregiver environment, standardized technology and safety procedures are essential and have been formalized in RS-tDCS protocol guidance [54].

More broadly, IoMT systems can enhance NIBS delivery by digitizing session-level metadata, including dose, impedance, tolerability, and timing relative to training, and linking these data to contemporaneous performance and physiologic metrics obtained from wearables or rehabilitation robots [8,18,28,30,31,33]. This integration provides a clearer understanding of adherence and response over time and supports the development of closed-loop NIBS, in which stimulation scheduling or intensity is adjusted based on objective indicators such as daily activity, sleep patterns, plateauing task performance, or physiologic signs of fatigue inferred from movement quality and load [18,33].

### 4.2. Functional Electrical Stimulation

FES delivers patterned electrical stimulation to peripheral nerves or muscles to elicit or augment voluntary movements, such as peroneal-nerve stimulation for dorsiflexion during ambulation and upper-limb stimulation to support grasp-related tasks [10,56,57]. Contemporary post-stroke FES systems include both open-loop (therapist- or patient-triggered) approaches and closed-loop paradigms that trigger stimulation using physiologic or intent signals such as surface EMG, kinematics, or BCI outputs [56,58,59]. Systematic reviews of upper-limb FES report improvements in motor impairment and functional task outcomes across various device configurations, but highlight substantial heterogeneity in stimulation dosing, triggering strategies, comparators, and outcome measures which complicate generalizability and protocol standardization [56,60]. Peroneal-nerve FES has demonstrated evidence of improving gait outcomes when combined with physiotherapy, although effect sizes vary across studies [57].

IoMT-enabled FES devices can record stimulation parameters, such as pulse amplitude, width, and frequency, as well as usage patterns and, in some cases, surface EMG or kinematic signals, which are uploaded for remote monitoring and titration [9,10]. Prototypes that integrate EMG sensors, FES, and virtual-reality environments illustrate closed-loop architectures in which stimulation timing and task difficulty are adapted in real time-based performance metrics [10,28]. This approach enables personalized progression of task complexity, automated adherence tracking, and timely clinician intervention when usage or response declines. However, practical barriers remain, including device complexity and the training burden for patients and caregivers.

### 4.3. Robotic Systems

Robot-assisted rehabilitation enables high-intensity, repeatable, task-specific practice while providing objective quantification of kinematics, kinetics, and active participation through embedded sensors [28,29]. IoMT-connected robots can transmit session-level metrics, including frequency, repetitions, assistance levels, and trajectory and force profiles, to secure platforms to support remote supervision and longitudinal progression [8,30,31]. A clinic-to-home feasibility framework using a planar rehabilitation robot demonstrates how structured remote programs can be implemented with minimal therapist oversight and regular performance logging [11].

In addition to feasibility studies, a prospective cohort study of a mirror-therapy rehabilitation robot applied to both upper and lower limbs reported improvements in motor and functional outcomes compared with conventional rehabilitation, supporting the clinical plausibility of robotic paradigms that integrate augmented feedback with sensor-derived performance capture [32]. Hybrid approaches that combine robotics with FES or NIBS are of particular interest because they can synchronize stimulation with specific phases of movement quantified by robot or wearable sensors. This design principle aims to strengthen use-dependent plasticity by coupling intent, stimulation, and executed movement [10,28,33,58].

## 5. Patient Interfaces

Although in-person assessment and care remain the gold standard of stroke neurorehabilitation, challenges regarding their accessibility persist, such as limited access to nearby rehabilitation providers, dose limitations of in-person sessions, and traveling difficulties—particularly for patients dealing with new neurological deficits [61]. The advancement of telemedicine technology and implementation has allowed patients to have greater access to rehabilitation resources. Electronic interfaces help meet these challenges by streamlining the patient’s transition from initial hospitalization to outpatient or home rehabilitation, producing better access and continuity of care [61]. Innovations like BCI, VR, and telerehabilitation present alternatives and supplementation to conventional stroke rehabilitation, allowing continuous monitoring without the constraints of space, and potentially provide more accurate measurements of functional metrics within the patient’s home environment [62,63,64,65]. In the following sections, this paper highlights the different approaches in neurorehabilitation underpinning these three innovations.

### 5.1. Telerehabilitation

Research on stroke telerehabilitation has increased in the aftermath of the COVID-19 pandemic, during which in-person services were reduced or shut down entirely [62,64,66]. Stroke telerehabilitation is the delivery of stroke therapy through remote communication methods, which may be synchronous, e.g., video conferencing, or asynchronous, e.g., watching a video within a phone application [61,62]. Most modalities involve some kind of guided therapeutic exercise, and services are targeted to improve motor or daily function, communication, depression, or stroke risk factors [62,67,68]. Telerehabilitation can contain gamified elements [61,65]. For example, a clinician may select therapeutic exercises on a gaming console for the patient and monitor their completion during or after completion [67]. The underlying approach behind gamification in neurorehabilitation is to activate dopaminergic pathways associated with the ventral striatum, the brain region responsible for reward processing and motivation, and thus promote a stroke therapy approach that is engaging, motivating, and easily accessible while still being highly intense and task-oriented [61,65,69]. Telerehabilitation has been shown to improve post-stroke impairments, disability, and patient quality of life, while reducing depression in patient caregivers [62]. Studies have shown similar or even better outcomes for patients receiving telerehabilitation compared to conventional stroke therapy [61,62,63,67,68]. The major benefits posed by telerehabilitation are time- and cost-savings, especially for patients living in areas with limited healthcare access [63]. Conversely, telerehabilitation may be more time- and cost-consuming for such patients as remote communication often requires reliable electricity, access to video capable devices, and mobile phone network coverage, which may create challenges to access [63]. Technical proficiency and general computer literacy on the part of the patient or their caregiver may be barriers as well [63,65]. Devices requiring specific setup sequences may be especially difficult for stroke patients who are already struggling with motor or cognitive impairments, and the lack of in-person clinical supervision or correction during asynchronous sessions may limit patient compliance with telerehabilitation [65,70,71].

### 5.2. Virtual Reality

Much like telerehabilitation, VR is a rapidly expanding field in stroke rehabilitative therapy. VR is a broad classification commonly divided into immersive and non-immersive modalities [65]. Immersive VR is generally characterized by user interaction with a completely virtual environment, usually through a VR headset, while non-immersive VR generally presents the virtual environment on a monitor, with which the user interacts using a controller, as in gaming consoles [65,69]. VR is derived from video games, and as with telerehabilitation interventions, gamified elements are often seen [69]. Studies primarily show benefits in upper extremity motor recovery, especially with immersive VR and in combination with conventional therapy [61,68,69,72,73,74]. A recent meta-analysis also found that VR game therapies were more effective than conventional therapy in improving cognitive ability, attention, mobility, and emotional state [73].

Generally, the major benefit of gamified interventions like VR is greater patient engagement and subsequent compliance, but these factors themselves depend greatly on personal factors such as the patient’s intrinsic motivation; successful outcomes require the patient to link their extrinsic in-game rewards to intrinsic goals of improving their neurological deficit [65]. Furthermore, as with telerehabilitation, technical competence poses a major barrier to VR therapies, as they require knowledge and potentially advanced training both for the patient and the clinician [65]. Cost may also play a prohibitive role in the initial purchase of VR equipment [65]. A unique challenge with the adoption of immersive VR therapies is the adverse effect of motion sickness [69]. The same effect is also associated with non-immersive VR, albeit reduced, which may make it more appropriate for routine clinical use [69].

### 5.3. Brain–Computer Interface

Broadly speaking, stroke rehabilitation therapies aim to potentiate neuroplasticity with the goal of maximizing nervous system functions after brain injury [10]. For example, motor rehabilitation in conventional stroke therapy may involve patients performing a core-stability exercise in order to strengthen and create new neural connections between the intent and the execution of such an exercise [64]. Enhancing this neural rewiring by closing the “central-peripheral-central” loop of intent, execution, and feedback is the basis of BCI [75]. In BCI, the patient is connected to an electroencephalogram (EEG) and is asked to attempt a task [61]. As the patient thinks about this activity, their central nervous system is activated, generating neural activity linked to the intention of task performance [76]. The EEG captures this neural activity, and the BCI reads the brain signals to provide the patient with some kind of feedback, which may be visual (an image of the task being completed), robotic (a device they wear which helps complete the task), or so on [61]. This bypasses brain lesions that prevent stroke patients from activating their peripheral nervous system during task execution, and returning feedback to their central nervous system upon task completion [61]. BCI is an emerging field that has applications in motor, cognitive, and emotional regulatory rehabilitation post-stroke [77]. Studies have shown its effectiveness is comparative to conventional stroke therapy, especially for upper extremity motor functions measured by the Fugl–Meyer assessment [61,75,78,79]. There is also evidence that BCI can improve attention and other cognitive functions in stroke, although its use in cognitive rehabilitation is more well-studied in non-stroke populations, such as patients with attention deficit disorder [61,79]. BCI interventions are often linked to motor imagery, a neuroscience concept and therapy which promotes neuroplasticity by activating the motor cortex with the imagery of a motor task instead of motor execution [61,79]. With the advent of AI, integration of BCI with machine learning may improve neural activity decoding capabilities and allow BCI therapies to provide more tailored feedback to patients [77]. To this extent, the adoption of BCI for clinical use may present new challenges for data privacy and liability due to the sensitive and personal nature of the data it uses and the feedback it recommends. Other limitations associated with BCI are mainly related to its technical complexity: clinicians and patients need to be trained, and patients in particular must undergo potentially lengthy calibrations to ensure proper functioning of the closed-loop system [75,80]. The calibration process itself may be hindered if the patient’s post-stroke deficits in the motor cortex are too severe, as it creates difficulties with detecting motor intention [75].

## 6. Data Processing

As rehabilitation technology has moved toward more data-driven care, the collection and interpretation of patient information have changed dramatically. One of the biggest shifts has been remote access to real-time physiological and biomechanical data. Several studies have shown that remote sensors allow for effective monitoring of therapeutic exercises and functional performance outside the clinic [9,81]. This is especially important for patients with neurological conditions because progress often depends on regular monitoring and accurate feedback [82]. With information from inertial sensors, gyroscopes, accelerometers, and pressure sensors, clinicians can evaluate gait and physical progress in the rehabilitative phase [9]. Additionally, physiological data can also be monitored through the use of EMG, ECG, and EEG to better assess recovery. However, data from these sources require extensive processing to extract relevant data from the wealth of information transmitted. Band-pass and notch filtering techniques may reduce motion and electrical artifacts, while sensor fusion or drift correction can reduce errors in quantification of linear acceleration, angular velocity, and orientation [83]. For biomechanical data, low-pass Butterworth filters are frequently used to reduce high-frequency noise while preserving meaningful kinematic signals [84]. Similarly, EEG data require artifact removal methods, such as independent component analysis, to remove aberrant readings from eye blinks, muscle activity, and motion before reliable interpretation or model training is possible [85].

Remote monitoring also allows earlier detection of problems. A stroke patient with subtle changes in range of motion or tone can be detected with remote monitoring long before an in-person appointment [82]. Remote activity-sensing aftercare programs have already demonstrated the value of continuous monitoring [86]. As rehabilitation devices such as robotic systems, BCI-controlled interfaces, and sensor-embedded orthotics become more complex and widespread, remote clinician oversight can improve efficiency, safety and continuity of care. However, storing and processing remote data comes with many challenges including fragmented data ownership and inconsistent data handling. Raw sensor data often is stored by device manufacturers, while processed summaries are stored by health care systems, making it difficult to share and integrate information across platforms [87]. Furthermore, differences in how devices measure and define signals can complicate integration of data between systems [87]. Open-source middleware and standardized data frameworks, such as Open mHealth, address this challenge by providing standardized ways to represent time-series health data, which allows heterogeneous sensor outputs to be mapped to consistent clinical measures [46]. Research has shown that open-source, community-built tools to aggregate data from commercial devices improve transparency and interoperability in the clinical setting [46].

Although standardization improves interoperability, it does not eliminate the need to process large volumes of continuous sensor data into clinically meaningful information. Consequently, data processing has become an integral bridge between raw data and actionable clinical insight.

Machine learning (ML) is a major contributor to rehabilitation data processing as it helps clinicians make sense of the enormous amount of data from patients. Some of the common supervised learning algorithms used in ML include support vector machine, decision tree, random forest, and artificial neural network [9]. For sequential data, algorithms such as recurrent neural networks, long short-term memory networks, and temporal convolutional networks are better at modeling the time-dependent nature of the recovery process [88]. By using algorithms that can detect complex patterns across large datasets, ML models can assist clinicians in predicting recovery trajectories, personalizing treatment plans, and identifying subtle markers of improvement or decline. In many cases, ML models can identify patterns in sensor and clinical data that humans would be unable to detect. For example, ML has been used to classify rehabilitation movements and evaluate movement quality using wearable sensors [81]. ML models can also consistently detect proprioceptive deficits in stroke patients [89]. Some studies also suggest they may help predict recovery patterns and, in certain cases, identify stroke subtypes [89]. Similarly, models using robotic-assisted rehabilitation data have classified stroke severity with high accuracy [90]. Therefore, ML may eventually have a diagnostic role to supplement current imaging techniques.

Another promising area is predictive modeling in neurorehabilitation. ML systems have already shown success in predicting early upper-limb recovery trajectories and functional outcomes after spinal cord injury rehabilitation [91,92]. Additionally, a study comparing COX regression (linear) models against ML-based (non-linear) models found the latter superior in predicting gait recovery following ischemic stroke [93]. The additional information from ML systems may enable clinicians to better structure recovery schedules and personalize therapy goals.

**Figure 2:**
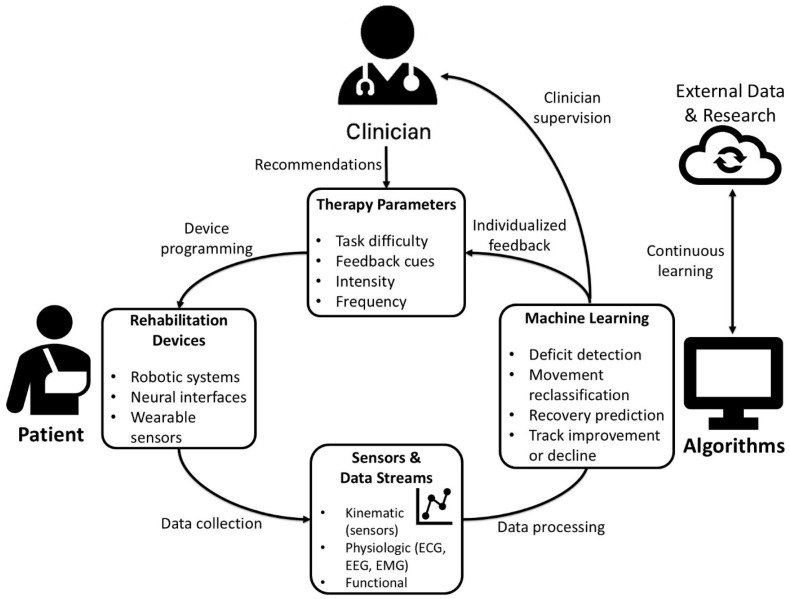
Closed-loop schematic of machine learning for neurorehabilitation (Legend: ECG—electrocardiogram; EEG—electroencephalogram; EMG—electromyography). @Brandon Khanyan via PowerPoint.

However, there are many limitations. Datasets for neurorehabilitation patients are often small and highly individualized, making it harder for models to generalize information [94]. Conversely, applying these models in clinical practice is often prone to overfitting in stroke populations where every lesion is unique. As a result, researchers are exploring strategies like transfer learning and synthetic data generation, which let models leverage information from larger or related datasets [92]. Federated learning is another promising approach that allows hospitals to train shared models together without actually sharing patient data [95]. This helps overcome both the challenges of limited datasets and privacy concerns. Clinicians also need models that are easy to interpret, so the information can be safely adapted into patient care and approved by regulatory bodies [94]. Unfortunately, more complex and better performing models are less intuitive and transparent with the decision-making process [96]. This necessitates the use of explainability techniques such as SHAP and LIME to help decipher why the algorithms suggest specific recommendations [96]. Ultimately, the goal is to combine multiple data streams including physiological, environmental, and behavioral data to assess a patient’s recovery. These systems also have the capability to continuously learn from new patients, gradually improving the effectiveness of machine assistance in neurorehabilitation (Figure 2).

## 7. Case Studies of Integrated Systems

Advances in telemedicine, closed-loop neurorehabilitation, and AI are transforming recovery by enabling continuous, data-driven therapy that extends seamlessly from clinic to home.

### 7.1. Telemedicine and Virtual Rehabilitation Networks

Telemedicine and virtual rehabilitation networks have been particularly effective in expanding access to high-intensity rehabilitation beyond traditional hospital settings [97]. For instance, the large phase III TRos/TR-2 Trial demonstrated improved upper-limb recovery in subacute stroke patients through a program delivering 70 min per day of home-based arm training, using games, exercises, and educational content over six to eight weeks [98]. Another example, the six-month ACTIV Trial, utilized a hybrid approach combining minimal in-person visits with text and phone support, leading to significant improvements in physical function for participants who adhered to the program [99]. The effectiveness of these virtual approaches is further highlighted by the VA Telehealth Programs, which include the Remote Patient Monitoring-Home Telehealth (RPM-HT) initiative to monitor post-stroke function and chronic risk factors, and the Telestroke Program, which provides real-time neurologist access for acute care in rural facilities [100]. However, a critical limitation in many current telerehabilitation studies, including trials like TRos/TR-2 and ACTIV, is the typically short follow-up window. This presents an overly optimistic view of adherence rates. The “drop-off effect” or “rehab attrition” is a well-documented phenomenon in chronic care, where initial high engagement with gamified VR or AI-coaching solutions often plummets after the first month due to psychological fatigue and lack of sustained motivation [101,102]. Future research must shift focus toward developing mechanisms for maintaining long-term adherence, potentially by integrating adaptive coaching models, peer support networks, or personalized motivational feedback that extends well beyond the initial subacute phase of recovery.

### 7.2. Closed-Loop Systems for Motor Recovery

Closed-loop systems represent a significant leap in neurorehabilitation by integrating sensors, stimulators, and adaptive algorithms to provide real-time therapeutic adjustments. A primary example is the BrainGate2 Trial, which utilizes a brain–computer interface (BCI) to convert motor intent directly into robotic arm movements. This technology enables individuals with paralysis to perform goal-directed actions, demonstrating the profound potential for neuroplastic motor restoration through direct neural engagement [103,104]. It is crucial to note, however, that BrainGate2 represents a highly experimental, laboratory-based intervention requiring invasive surgical implants and continuous technical oversight from research engineers to maintain signal stability [105]. In contrast, commercial systems bridging the “Laboratory-to-Living-Room” gap often rely on non-invasive hardware. Similarly, the MindMotion™ PRO System provides a CE-certified VR platform that uses motion capture and gamified feedback to reinforce correct movement patterns. Its closed-loop design is specifically engineered to adjust task difficulty continuously based on a patient’s immediate performance, a feature that actively encourages neuroplastic adaptation. Clinical evaluations of the system have shown significant improvements in Fugl-Meyer Assessment scores and overall patient motivation [106,107,108]. Together, these cases highlight how the delivery of real-time sensorimotor feedback can accelerate the recovery process while maintaining high levels of patient engagement.

### 7.3. Artificial Intelligence and Data-Driven Personalization

AI has become a cornerstone of modern neurorehabilitation by enabling the automated tailoring of therapy intensity, content, and progression. This shift toward data-driven personalization is exemplified by the AISN Trial, a multicenter study testing an AI-driven decision-support module within the RGS@home VR system. By personalizing task difficulty based on real-time performance, this AI optimization aims to significantly improve motor recovery compared to traditional methods [109,110]. Similarly, the AI Cognitive Telerehabilitation Trial utilized the AI-guided Zenicog^®^ platform, demonstrating that automated systems can perform comparably to therapist-supervised cognitive training [12].

Beyond software, AI-guided robotics such as the Ekso exoskeleton and IpsiHand device use machine learning to analyze EMG and EEG signals, allowing for highly individualized motor training by adjusting physical assistance levels in real time [111,112]. A critical consideration, however, is the necessity of robust “Safety-First” frameworks. When AI algorithms interpret bio-signals (like EMG/EEG), a risk of “algorithm hallucination” exists, where a stray signal is misinterpreted as user intent, potentially resulting in an unintended or jerky movement and a risk of patient fall [113]. To mitigate these physical risks, all clinical robotic systems are mandated to integrate fail-safe mechanisms, including prominent, easily accessible physical “kill-switch” protocols (emergency stops) that immediately cut power to the device [114]. Furthermore, the development and deployment of these devices are governed by stringent regulatory standards (e.g., FDA, CE-certification) that establish liability frameworks and mandatory risk assessments to ensure patient safety remains paramount during AI-driven physical assistance [115]. AI-enhanced VR systems like VRehab, NeuRRoVR, and MindMotion GO further support this trend by using multimodal sensors to modify task complexity dynamically and deliver automated feedback [116,117,118]. This technological integration extends to AI-powered mobile applications, such as those developed by the University of Texas Health Houston for exercise coaching or iTalkBetter for language rehabilitation, which analyze movement and speech to provide immediate clinical guidance. Across all these domains, AI effectively increases therapy dosage, precision, and personalization, ensuring that rehabilitation is both intensive and uniquely suited to the patient’s needs.

### 7.4. Synergistic Integration

The future of rehabilitation lies in the synergistic integration of telemedicine, closed-loop devices, and AI into cohesive, end-to-end ecosystems. One such example is the Motor Recovery Ecosystem, which combines EMG-triggered FES wearables and motion sensors with AI engines to create an adaptive home-based environment. Monitored via telerehabilitation dashboards, this system continuously predicts recovery trajectories and dynamically adjusts exercise intensity, providing high-intensity retraining that blends independent practice with professional oversight [82]. Similarly, the Cognitive Rehabilitation Ecosystem utilizes lightweight EEG headsets to monitor neural markers of attention and fatigue in real time. This allows a sophisticated AI engine to automatically adjust task complexity or trigger neurostimulation, such as tDCS, ensuring the patient remains in an optimal learning zone while neuropsychologists review analytics through a dedicated portal [119,120].

This data-driven approach extends to physical mobility through the Gait Rehabilitation Ecosystem, where wearable IMUs feed data into AI that modulates smart orthoses or exoskeletons. This provides immediate correction of gait asymmetry and enables remote physical therapy reviews, shifting the paradigm from episodic clinical visits to immediate, daily gait correction [121]. It is important to acknowledge that the “Ecosystems” described above represent an aspirational vision rather than a universally interoperable reality. A significant current technical barrier is the “Data Silo” problem: many current devices use proprietary APIs and encrypted data formats that prevent seamless communication. The realization of true end-to-end solutions requires the development and industry-wide adoption of unified IoMT Communication Standards to ensure that IMU data, AI analytics, and smart orthoses can reliably “talk” to one another [122]. Communication recovery is also being transformed by the Aphasia Rehabilitation Ecosystem, which integrates mobile technology with AI-driven natural language processing. This system monitors speech metrics like fluency and word retrieval in real time, immediately adjusting exercise difficulty while Speech-Language Pathologists review “speech heatmaps” to guide long-term progress [123,124].

Finally, the Chronic Pain Precision Ecosystem utilizes biosensors to detect early pain flare signatures. This allows predictive AI algorithms to trigger closed-loop neuromodulation, such as spinal cord stimulation or vagus nerve stimulation, before symptoms escalate. By allowing a telemedicine team of pain specialists to review weekly AI reports and refine medication protocols, these integrated ecosystems demonstrate how continuous data flow can deliver highly personalized, intensive therapy within the daily lives of patients [125,126].

## 8. Discussion

Stroke remains a leading global cause of long-term disability, causing substantial personal, social and economic burdens, with patients in under-resourced nations being most affected. Conventional rehabilitation remains the foundation for post-stroke recovery, but its effectiveness is often constrained by accessibility and long-term engagement. The IoMT provides a unifying infrastructure capable of addressing some of these limitations by enabling continuous monitoring, data management and analysis, data-driven personalization, and scalable delivery of rehabilitation. IoMT-enabled sensors, wearables, robotic systems, and telemedicine platforms allow objective, high-frequency capture of physiological, motor and cognitive data in real-world environments. When combined with cloud computing and ML, these data streams support adaptive, closed-loop rehabilitation models that individualize medical care, optimize timing of interventions such as FES or NIBS, and enable early detection of plateaus or decline. Emerging evidence from randomized trials, cohort studies, and feasibility frameworks indicates that such approaches can achieve outcomes comparable to, and in some cases exceeding, conventional rehabilitation therapy, while expanding access for patients in resource-limited or remote settings.

The combination of HL7 FHIR and IoMT can be highly effective in bridging the digital divide in low-resource settings by utilizing technologies such as asynchronous transmission (store-and-forward and opportunistic networks) to handle intermittent connectivity, edge/fog computing to conserve bandwidth and lengthen device life, FHIR resources for semantic interoperability across platforms, security mechanisms scalable to resource constraints, and hybrid architectures that combine local processing with cloud synchronization when available. Low-resourced settings often have intermittent or low-bandwidth connectivity (e.g., cellular, opportunistic networks), prohibiting constant online access on which cloud models rely. Intermittent connectivity handling and asynchronous data forwarding help increase reliability in environments where continuous internet access is not guaranteed [127]. Traditional IoMT systems that send raw data continuously to the cloud consume significant network bandwidth and quickly drain wearable batteries. Edge computing processes and filters data on or near the device, reducing bandwidth use and allowing for real-time monitoring even with unstable network infrastructures [128]. These strategies help overcome infrastructure limitations in low-resourced locations while maintaining interoperability and clinical relevance to the local population.

A key challenge in deploying advanced neurorehabilitation technologies in under-resourced settings is the apparent contradiction between the global burden of neurological disability and the infrastructure and cost typically associated with NIBS, BCIs, and robotic systems. Frugal IoMT utilizes approaches that emphasize low-cost wearable sensors, mobile devices, local (edge) data processing, and asynchronous data transmission over widely available 3G/4G networks. Evidence shows that clinically meaningful neurophysiological and kinematic data can be processed locally and transmitted intermittently, reducing bandwidth, power, and infrastructure requirements while maintaining effectiveness [129]. Portable, battery-powered NIBS devices, simplified BCI pipelines using on-device computation, and modular, low-cost robotic or sensorized rehabilitation systems have all demonstrated feasibility outside high-income clinical environments [130]. When integrated into a frugal IoMT architecture, these technologies can operate autonomously with minimal connectivity, transmitting only summary metrics or alerts, thereby supporting scalable, context-aware neurorehabilitation. Collectively, this paradigm demonstrates that advanced digital rehabilitation ecosystems can be adapted for low-resource health systems and are not inherently “luxury solutions” limited to the developed world.

As neurorehabilitation transitions toward home-embedded IoMT ecosystems, human-in-the-loop ethical frameworks are essential to ensure that caregivers remain supported rather than overburdened. Incorporating caregivers as active stakeholders in system design and evaluation is therefore critical to ensuring that IoMT solutions genuinely reduce, rather than redistribute, rehabilitative work. Similarly, variability, challenge, and occasional failure are essential for durable skill acquisition in rehabilitation. Accordingly, AI-guided training should be designed within human-in-the-loop frameworks, allowing therapist oversight to balance algorithmic adaptation with experiential learning and clinical intuition. Further, the integration of IoMT with virtual reality, brain–computer interfaces, robotics, and AI-driven decision support illustrates a broader paradigm shift from episodic, clinic-centered rehabilitation toward continuous, home-embedded recovery ecosystems. These systems emphasize patient engagement, neuroplasticity-driven training, and longitudinal outcome tracking, aligning rehabilitation more closely with the principles of precision medicine. However, significant challenges remain, including further technological development in data interoperability, cybersecurity, regulatory oversight, algorithm interpretability, and the need for large, diverse datasets to support generalizable machine-learning models.

## 9. Conclusions

Finally, IoMT-based neurorehabilitation represents a transformative opportunity to enhance stroke recovery by increasing therapy intensity, personalization, and accessibility while maintaining clinical oversight. The challenge of bridging the digital divide and the provision of the latest neurorehabilitation modalities to low-resourced communities have to be balanced with the further development of frugal IoMT architectures that highlight function and design within constrained settings. Future progress will depend on rigorous multicenter trials with standardized outcomes, harmonized data standards, and interdisciplinary collaboration among clinicians, engineers, and policymakers. Addressing these challenges will be essential for translating promising IoMT-enabled technologies from research settings into equitable, routine clinical care that improves functional independence and quality of life for the growing population of stroke survivors.

## Data Availability

No new data were created or analyzed in this study. Data sharing is not applicable to this article.

## Abbreviations

The following abbreviations are used in this manuscript:

AI	Artificial intelligence
BCIs	Brain–computer interfaces
ECG	Electrocardiogram
EEG	Electroencephalogram
EHR	Electronic health record
EMG	Electromyography
FES	Functional electrical stimulation
FHIR	Fast Healthcare Interoperability Resources
HL7	Heath Level Seven International
IMU	Inertial measurement unit
IoMT	Internet of medical things
MCID	Minimal Clinically Important Difference
ML	Machine learning
NIBS	Non-invasive brain stimulation
RS-tDCS	Remotely supervised-transcranial direct current stimulation
tDCS	Transcranial direct current stimulation
rTMS	Repetitive transcranial magnetic stimulation
VR	Virtual reality
